# Habit Discontinuity, Self-Activation, and the Diminishing Influence of Context Change: Evidence from the UK Understanding Society Survey

**DOI:** 10.1371/journal.pone.0153490

**Published:** 2016-04-27

**Authors:** Gregory Owen Thomas, Wouter Poortinga, Elena Sautkina

**Affiliations:** Welsh School of Architecture, Cardiff University, Bute Building, King Edward VII Ave, Cardiff, CF10 3NB, United Kingdom; Shandong University, CHINA

## Abstract

Repeated behaviours in stable contexts can become automatic habits. Habits are resistant to information-based techniques to change behaviour, but are contextually cued, so a change in behaviour context (e.g., location) weakens habit strength and can facilitate greater consideration of the behaviour. This idea was demonstrated in previous work, whereby people with strong environmental attitudes have lower car use, but only after recently moving home. We examine the habit discontinuity hypothesis by analysing the *Understanding Society* dataset with 18,053 individuals representative of the UK population, measuring time since moving home, travel mode to work, and strength of environmental attitudes. Results support previous findings where car use is significantly lower among those with stronger environmental views (but only after recently moving home), and in addition, demonstrate a trend where this effects decays as the time since moving home increases. We discuss results in light of moving into a new home being a potential ‘window of opportunity’ to promote pro-environmental behaviours.

## Introduction

Transportation is the second largest producer of greenhouse emissions in the UK [1]. Cars and taxis are the largest contributor of these emissions [2], and given that 77% of the total distance travelled by UK commuters is by car [3], travel mode choice has serious environmental implications. To encourage more sustainable actions, we may seek to understand the causes of behaviours, and travel mode choice has been a topic of interest for policymakers and researchers alike. Some work has understood travel mode choice as the conscious processing of personal views and circumstances that lead to an intention to choose a particular travel mode, most famously by the Theory of Planned Behaviour [TPB; 4]. The TPB posits that behaviour is the result of a conscious intention to enact the behaviour, and that this intention is formed by people’s attitudes to the behaviour, social norms toward the behaviour, and perceived behavioural control, which reflects the ability to enact the behaviour [4]. The TPB has proved extremely popular in travel mode choice research, and a review by Gardner and Abraham [5] showed by a meta-analysis of 29 studies that intentions can predict about 28% of variance in travel mode choice.

### Habits and Habit Discontinuity

Alongside the strengths of the TPB, Gardner and Abraham [5] reported that habitual processes explain an almost equal amount of variance in behaviour. Habits are generally defined as “*a form of automaticity in responding that develops as people repeat actions in stable circumstances*” [6, p. 91]. Although behaviour may originally be predicted by conscious intentions, over time an automatic association between the context and the behaviour forms so that behavioural intentions becomes less predictive of actual behaviour [7,8]. The interaction between habits and intentions has been demonstrated in several domains. A meta-analysis of 47 experiments highlight that intentions are weaker predictors of behaviour in stable contexts [9]. A subsequent meta-analysis of 9 studies found that habit strength mediated the link between intentions and behaviour [10]. Notably for travel mode choice, the stronger the habitual impulse to use a car, the weaker the link between intentions to use alternative travel modes and actual behaviour [11].

A complication with habits is the difficulty in changing them. Habitual behaviours are characterised by automatic processes, which may prevent people considering information to change their behaviour. In a series of experiments, stronger habitual travel mode choice was linked to fewer requests of available information to make a travel mode decision, even in novel circumstances [12,13]. This means that behaviour change campaigns that provide information to change intentions (and thus behaviour) are less effective for habitual behaviours [9], and sustainable transport campaigns may face difficulty in only using information to alter habitual travel patterns that are not attentive to new information [14].

A solution to the fixedness of habitual behaviours may lie in a defining characteristic of habits; they are contextually-cued, and thus dependent on a stable context [6,7]. Verplanken and Wood [6] therefore theorised that habit strength may be weakened, or even broken, by a sufficiently large change in the context that a behaviour is performed. This has become known as the habit discontinuity hypothesis [15]. Support for changes in context to weaken habitual thought processes has been demonstrated in several areas, including economic game simulations, where changing task scenarios improved the facilitation of new information and rejection of past routines [16,17]. Reliance on context was demonstrated in an experiment involving stale popcorn and habitual strength of eating popcorn in cinemas. When seated in a cinema (conventional context), participants with strong popcorn habits ate far more stale (and unappetising) popcorn than those with weaker popcorn habits. But this pattern was not found when eating stale popcorn in a dark room, where the context was not linked to the habit, so there was no contextually-driven impulse to habitually eat unappealing popcorn [18]. Additionally, an field study of habit discontinuity was observed among staff at WWF UK, by measuring the habitual strength of using their travel modes prior to relocation of their workplace, 1 week and 4 weeks after office relocation [19]. Results indicated that all staff, independent of whether or not their travel mode changed after relocation, reported a reduction in the strength of their habitual choice of travel mode, demonstrating that the change in context weakened the automatic associations of context and behaviour.

### Habit discontinuity and the self-activation hypothesis

Verplanken et al. [15] not only found support for context change reducing habit strength, but also suggested that the discontinuity may be coupled with activating personal views. They highlighted research indicating that pro-environmental views did not necessarily predict pro-environmental choices; sustainable choices only become apparent after priming a person’s pro-environmental identity [20]. The authors proposed that because habits are weaker after a change in context, a re-assessment of the behaviour and situation may activate a person’s environmental views so that sustainable behaviour would be more likely for those with strong environmental views [15]. Verplanken et al. surveyed respondents to assess their mode of travel, environmental views, and the number of years since they had moved home as a measure of stable context. Results showed that recent home movers (<12 months) with stronger environmental views had lower levels of car use than those with comparably high environmental concern but who had not recently moved home, suggesting that the change in context facilitated this difference in travel mode choice [15].

The habit discontinuity hypothesis has a number of implications for promoting sustainable behaviour. Contextual changes that affect transportation occur frequently [21,22], both from unexpected (e.g., road closures) or planned changes (e.g., moving home). But to effectively inform interventions, additional investigation is required. Verplanken et al.’s [15] evidence for habit discontinuity and self-activation used a sample of 433 staff working at a university in England. Their sample was not representative of the population however: data came from a high-socioeconomic area, which may bias favourable responses to particular travel mode choices [23]. The built environment and area’s topography can affect sustainable travel choices [24], and the location of the university may also have influenced Verplanken et al.’s [15] results. Evidence for a combined habit discontinuity and self-activation hypothesis using a dataset from respondents across a wider range of geographic and social backgrounds would give more support to the research findings. This is particularly relevant with the current demand for replication and evaluation of concepts in psychology research: especially in social psychology [25,26].

In addition, our understanding of the timescale for how habitual behaviours form and change is limited. Measuring the automaticity of several daily behaviours, Lally and colleagues modelled the growth of habit strength for a selection of daily health behaviours [27]. There is also evidence on how the strength of a habit is reduced from a change in context, and then begins to become more automatic over time [19]. But we are not aware of any evaluation of how long a change in context (and thus habit disruption) may be linked to the self-activation hypothesis. In their investigation, Verplanken et al. [15] used a 12-month split of “recent” or “not recently moved” for analysis, a split that was acknowledged by the authors to be “arbitrary” (p. 123).

Therefore, this paper details an investigation into the habit discontinuity effect and the self-activation hypothesis. Seeking a more detailed and comprehensive sample than that used by Verplanken et al. [15], we used a large dataset representative of the UK population, the Understanding Society Survey (USS) [28].

### Aims of the Study

This paper has two research aims. First, we will see whether the time that a person has been living in their current residence is associated with changes in travel mode choice for commuting to work. In light of previous findings, we hypothesise that people who have recently moved home will show disruption in travel mode choice as previous habits are broken (Hypothesis 1). Second, we will include environmental attitudes as a predictor to see if there is a significant interaction between time since moving home and attitude strength. In accordance with habit discontinuity and self-activation, we hypothesise that a significant interaction will indicate that stronger environmental attitudes are a predictor of lower levels of car use, but only after recently moving home (Hypothesis 2).

## Method

The USS is a longitudinal panel survey representative of the UK population, sampling approximately 40,000 households on a variety of topics with interviews held every 1–2 years [29]. The USS replaces the British Household Panel Survey, the previous UK longitudinal panel survey that ran between 1991 and 2008. As well as measuring environmental attitudes and travel mode choice, the first USS data collection between January 2009 and March 2011 (termed Wave 1) asked participants how long they had lived at their current residence. Unlike more recent survey waves, Wave 1 of the USS uniquely asked this question for all respondents, as later waves only asked length of residence if they had newly joined the survey panel.

The USS dataset (6^th^ Ed.) was downloaded on 19^th^ January 2015. For all numbers reported, the cross-sectional weight for Wave 1 individual responses (*a_indscus_xw*) was applied to ensure the sample matched the UK population [29], and analysed using IBM SPSS v.20.

### Sample size & demographics

The total Wave 1 sample, with cross-sectional weight applied, totalled 39,986 individuals. Participants under the age of 17 (n = 1,388) were removed. The question of how people chose to travel to work was only asked if respondents indicated that they were employed or self-employed, and also that they worked in a location other than their own home. Of all respondents, 16,139 indicated that they were not employed or self-employed, and 12 responded that they didn’t know, and excluding these respondents resulted in a sample of 22,447 cases. For workplace locations, 1,763 people indicated that they worked from home, and were not asked the question on travel mode choice, which left a working sample of 20,684.

### Items within the USS

#### Respondent characteristics

Respondents specified their age and gender. The USS includes a derived variable of gross monthly income for each household based on several income sources (e.g., salary, benefits). The USS also describes whether a respondent lived in an urban or rural location. Socio-economic class used the NS-SEC method based on employment source, using the 3-group classification which may be treated as an ordinal measure [30]. The NS-SEC 3-group classification ranges from ‘Routine and never worked/long-term unemployed’, to ‘Intermediate’, and ‘Management and Professional’.

#### Environmental attitudes

Environmental attitudes were assessed using nine statements on climate change effects and willingness to act sustainably, measured on binary response of “I Believe/I Don’t Believe”, and these items are described in Table 1.

**Table 1:** Description of items measuring environmental attitudes.

#	Item Description
1	I don’t believe my behaviour and everyday lifestyle contribute to climate change
2	I would be prepared to pay more for environmentally friendly products
3	If things continue on their current course, we will soon experience a major environmental disaster
4	The so-called ‘environmental crisis’ facing humanity has been greatly exaggerated
5	Climate change is beyond control—it’s too late to do anything about it
6	The effects of climate change are too far in the future to really worry me
7	Any changes I make to help the environment need to fit in with my lifestyle
8	It’s not worth me doing things to help the environment if others don’t do the same
9	It’s not worth Britain trying to combat climate change, because other countries will just cancel out what we do

Scores of two items (2 and 3) with negative phrasing were reversed so that higher scores indicate stronger pro-environmental attitudes. The nine statements had a reliability of Cronbach’s α = .65.

#### Time spent at current address

**Fig 1:**
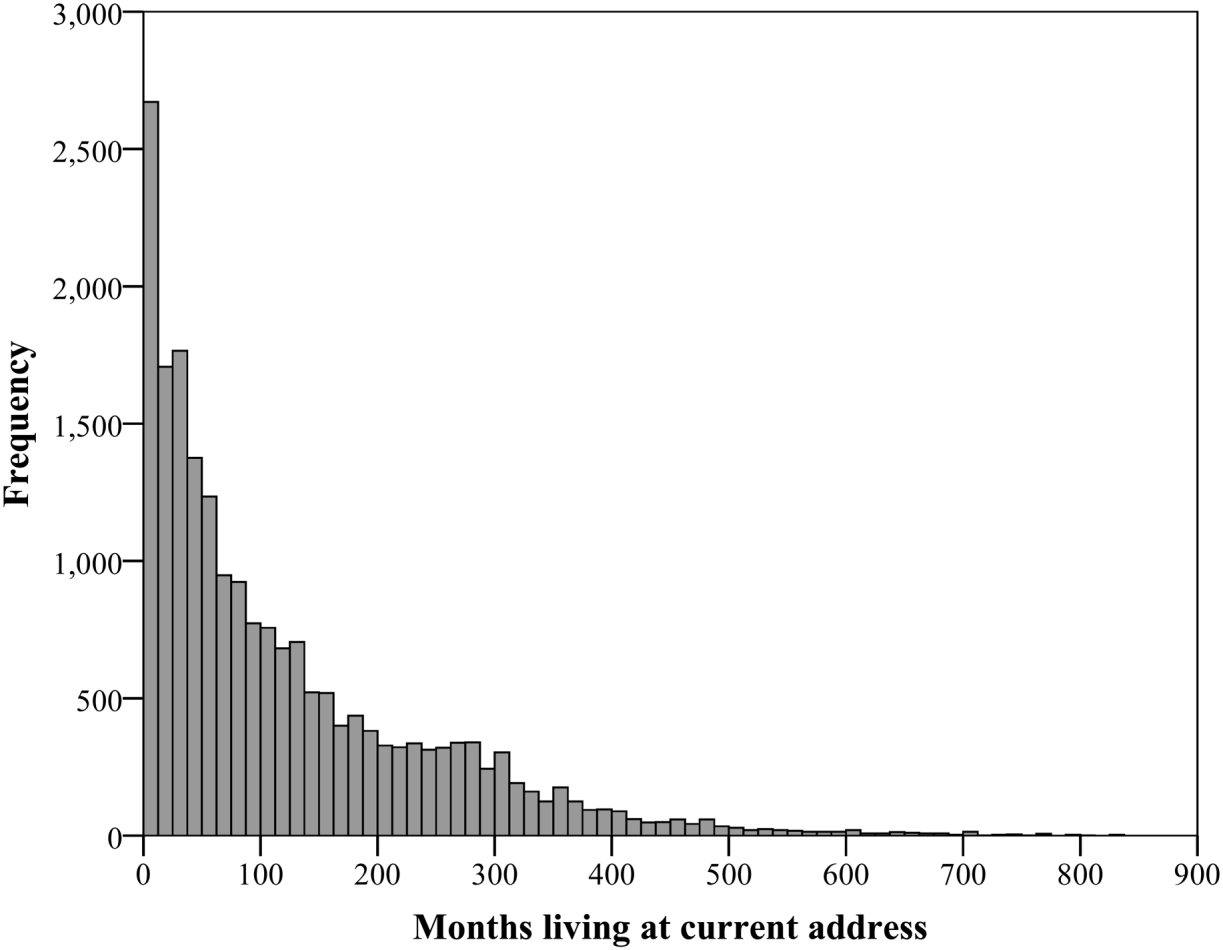
Frequency of responses by number of months that people have lived at their current address.

The USS recorded the date the respondent was interviewed, and date the respondent recalled moving into their current home (if they had not lived at that location their whole life), allowing the calculation of number of months since the person had lived at their current address. If respondents indicated that they had lived at their current address their entire lives, the date of the interview and the date of their birth were used to calculate the length of time lived at that location. Within the sample, time spent living at the current address ranged from 0 to 828 months (69 years), with a mean of 123 months (10 years and 3 months), and a median of 80 months (6 years and 8 months). The frequency of observations, by number of months living at the current address, approximates a logarithmic decay, *F* (1) = 6007.24, *p* < .001, R^2^ = .91, as shown in Fig 1.

**Fig 2:**
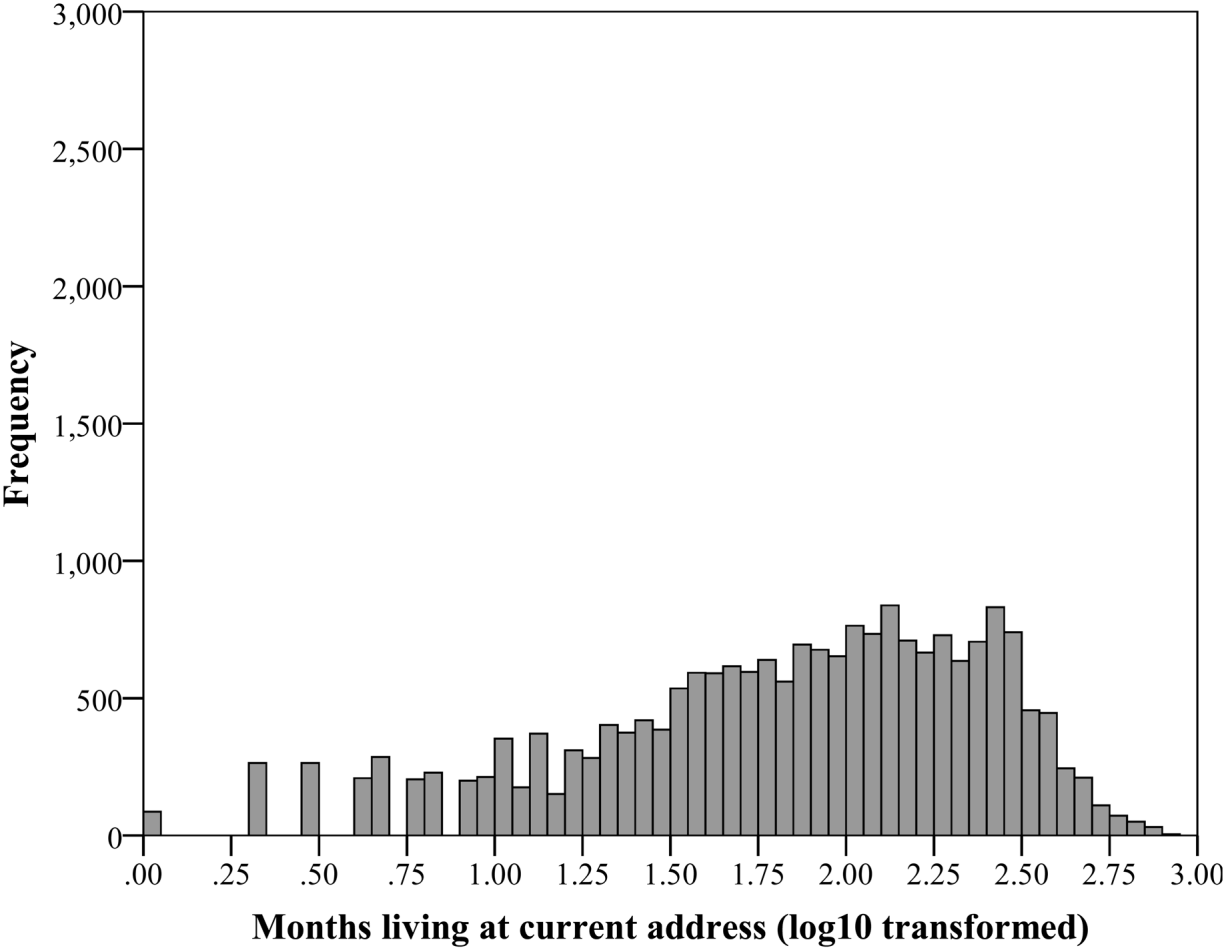
Frequency of responses by number of months that people have lived at their current address after log10 transformation.

As seen in Fig 1, the frequency of observations of time spent living at the current address shows a substantial positive skew. Extreme skews can cause complications for regression analysis, and when faced with a positive skew, a common approach is to apply a logarithmic transformation to the variable in question, which can substantially reduce skewness [31]. Therefore a log10 transformation was applied to the variable of time spent living in the current address, but with several cases indicating 0 months (which cannot be log transformed), it is advised to add a constant to each value before transformation [31], so the transformation for Time was log10(*x* + 1), where *x* was the value of time spent living at the current address for each respondent. Before transformation the skewness of the variable for Time was 1.54, and post-transformation the skewness was 0.82, with a revised histogram shown in Fig 2.

#### Travel mode choice

Respondents indicated their usual method of travelling to work from eleven transport choices. We separated these choices into two categories, 1) those travelling alone by a car or van, and 2) those travelling by alternative methods that included: getting a lift with someone within or outside their household, motorcycle/scooter/moped, taxi/minicab, bus/coach, train, underground/light railway/metro, cycling, walking, or undeclared ‘other’.

The majority of respondents used the car as their main travel mode (62.5%), compared to alternative modes (37.5%). Car users were 54.5% male, alternative mode users 49.6% male, a significant but negligible difference in gender, Χ^2^ (1) = 45.93, *p* < .001, Cramer’s V = .05. Car users (M = 42.0, SD = 12.3) were older than alternative mode users (M = 38.1, SD = 13.1), t (20,680) = 21.59, *p* < .001, indicating a “small” effect, Hedge’s *g* = .31. Gross monthly household income for car users (M = £4,344, SD = 2776.77) was higher than alternative mode users (M = £4,049, SD = 2805.80), *t* (20,608) = 7.37, *p* < .001, though with a very small effect, Hedge’s *g* = .11. Car users were slightly more likely to have a higher SES than alternative mode users; 44.9% were in management & professional roles compared to 37.1% of alternative mode users, Χ^2^ (2) = 279.69, *p* < .001, Cramer’s V = .12. Alternative mode users were also slightly more likely to live in urban areas, 87.0% compared to 74.8% of car users, Χ^2^ (1) = 441.99 *p* < .001, Cramer’s V = .15.

### Missing Value Analysis

Missing values presents an issue for analysis, as the results may be biased by a lack of responses for particular items. To check for missing values, Little’s MCAR (Missing Completely At Random) test was run on the demographic variables, environmental attitudes, time living at current address, and work travel mode. The test uses a chi-square distribution test to establish is there are patterns in the rate of missing values for items. Overall, Little’s MCAR test was significant, X^2^ (21) = 290.96, *p* < .001, indicating that data were not MCAR. This left two possibilities; missing data may be Missing At Random (MAR), or Not Missing At Random (NMAR). The former option is more desirable, as MAR data can be predicted from the measured variables, so we can be confident where the source of any potential bias lies. NMAR data implies that the source of missing data is unknown and may be related to the specific item and cannot be ignored [31]. Each variable was evaluated, and three were found to have missing data: SES had 107 counts of missing data (0.5% of all cases), environmental attitudes had 1,885 counts of missing data (9.1% of all cases), and time living at current address had 363 counts of missing data (1.8% of all cases). In a very large dataset, such as the USS, variables with <5% of missing cases are seen as less serious [31]. With 9.1% of cases missing for environmental attitudes, we tested to see whether missing cases were significantly different from observed cases using the demographic variables. Independent *t*-tests indicated that cases with missing data for environmental attitudes were generally older (t (20,682) = 16.96, *p* < .001, Hedge’s g = .41), had a lower SES (t (20,575) = 13.72, *p* < .001, Hedge’s *g* = .33), and had lower income (*t* (20682) = 10.77, *p* < .001, Hedge’s *g* = .26). In this circumstance, where missing data are not found in the DV (work travel mode choice), but are found in an independent variable, and that patterns in missing values can be determined from measured variables, the data may be assumed to be MAR, and not MCAR [31]. Given the substantial dataset, we chose to exclude missing cases from analysis, though missing values for environmental attitudes should be considered when interpreting results. After weighting, removing cases with missing data left 18,053 valid cases for analysis.

## Results

### Analysis 1: Time at current address

With a binary outcome (commute by alternative mode = 0, commute by car = 1,), binary logistic regression was used. First we included demographic variables of age, gross monthly income (“Income”), and socio-economic status (“SES”) as continuous variables. Variables of gender (0 = male, 1 = female), and living location (“Rural”, 0 = urban, 1 = rural) were categorical variables. We then added the log10 transformed time spent living at current address (Time). Results are highlighted in Table 2.

**Table 2:** Logistic regression results predicting commuting by car by time spent living at current address and demographic covariates. Final model fit *Χ*^*2*^ (6) = 1057.95, p < .001, Cox & Snell pseudo R^2^ = .06, Nagelkerke pseudo R^2^ = .08.

	B	S.E.	Wald	Exp (B)	Exp (B) 95%CI
Gender	-0.18	0.03	33.40	0.83***	(0.78 : 0.89)
Age	0.02	0.00	213.47	1.02***	(1.02 : 1.02)
SES	-0.21	0.02	130.41	0.81***	(0.78 : 0.84)
Rural	0.76	0.04	318.92	2.15***	(1.97 : 2.34)
Income	0.00	0.00	4.84	1.00*	(1.00 : 1.00)
Time	0.21	0.03	47.61	1.23***	(1.16 : 1.31)
Constant	-1.12	0.09	152.24	0.33***	

*** = *p* < .001,

** = *p* < .01,

* = *p* < .05.

Independent of gender, age, monthly income, socio-economic status and urban/rural location, it can be seen that the length of time living at one’s current address is a significant, positive predictor of commuting to work by car. Logistic regression calculates of the log odds of an event which may be difficult to interpret. To ease interpretation, log odds can be transformed into the predicted probability of the event occurring by using the following formula [32], where *x* is the log odds of commuting by car:

$$predicted\;probability=\;\frac{\text{exp}\left(x\right)}{1+\text{exp}\left(x\right)}$$

**Fig 3:**
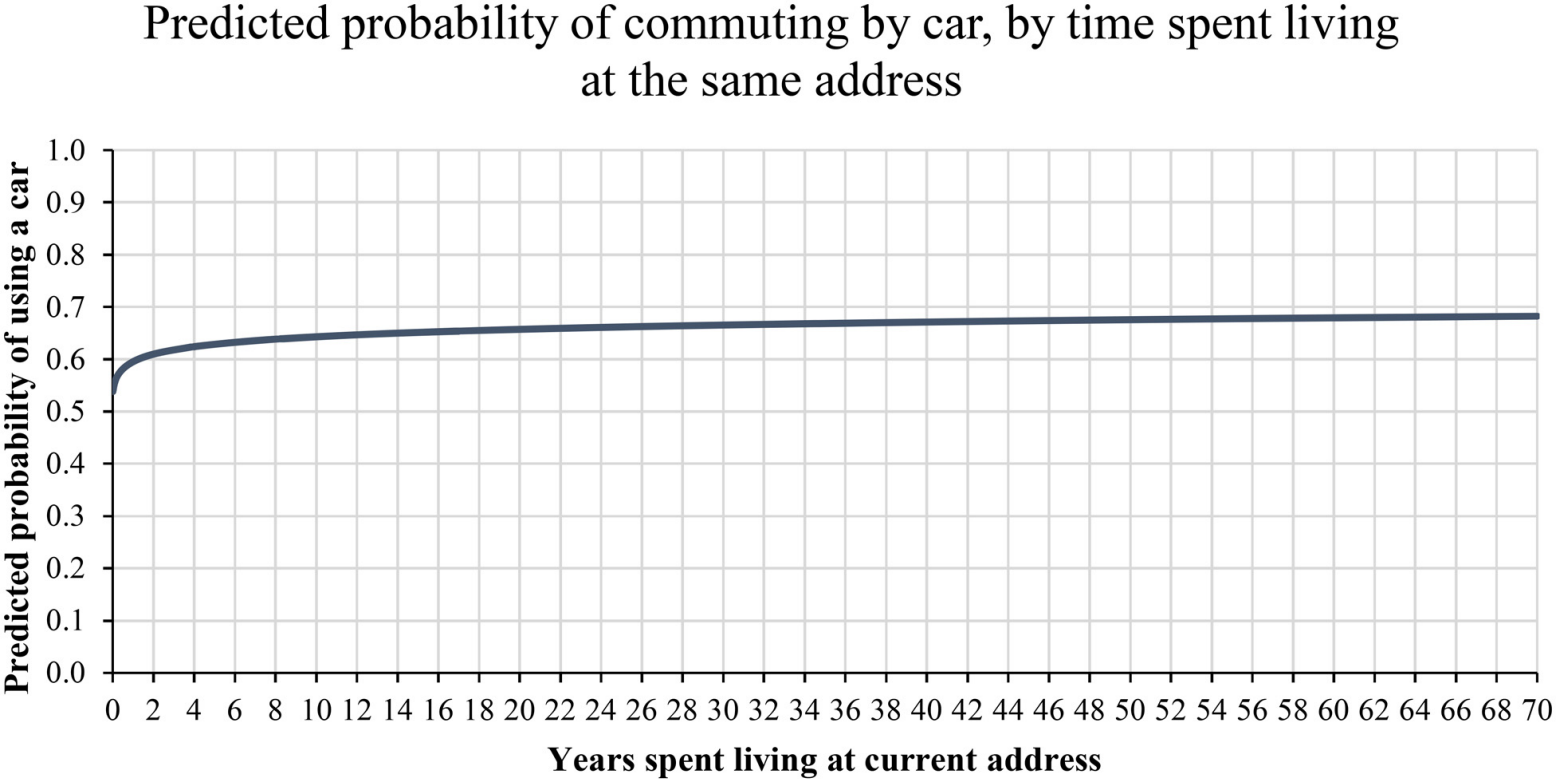
Predicted probability of commuting by car by time spent living at the same address, with covariates held at their mean values. Time was log10 transformed for analysis, and displayed here in the original metric for interpretation.

To illustrate the effect of time spent living at the current address on commuting travel mode choice, we plotted the predicted probability of commuting by car, as it varies by the coefficient of time spent living at the current address, whilst holding the coefficients of demographic covariates at their mean values. Results of this method are shown in Fig 3.

Results show that the time spent living at current address is linked to commute travel mode choice, particularly for those who have lived at the same location for only a short period of time (and most likely have recently moved). Holding the covariates constant at their mean, the regression model indicates that living at one’s current address for just 1 month is linked to a .54 predicted probability of commuting by car. As the time since moving home increases, the predicted probability also quickly increases, so that 12 months at the same address (again with covariates constant at their mean) is linked to a .60 probability of commuting by car. The rise in probability of car use then very slowly increases over time, with a predicted probability of commuting by car of .64 after 10 years in the same address. In support of Hypothesis 1, the dramatic change in probability for those who have recently moved home would suggest that there is a period of change where travel options are undergoing some uncertainty, and in line with the habit discontinuity hypothesis, may be open to stronger influence from environmental attitudes.

### Analysis 2: Time and attitude interaction

To extend the analysis, a binary logistic regression model was specified as before to predict commuting by car, with the addition of environmental attitudes (Attitude), and an interaction term between the time spent living at current address (log10 transformed) and environmental attitudes. The habit discontinuity and self-activation hypothesis would suggest that strong environmental attitudes would be a predictor of not commuting by car, but interacting with time spent at current address, so that attitudes are only linked to commute mode choice when a person has not lived in the same location for a long time. Results from the regression model are highlighted in Table 3.

**Table 3:** Logistic regression results predicting commuting by car by time spent living at current address, environmental attitudes, and their interaction effect (with covariates). Final model fit *Χ*^*2*^ (8) = 1122.49, *p* < .001, Cox & Snell pseudo *R*^*2*^ = .06, Nagelkerke pseudo *R*^*2*^ = .08.

	B	S.E.	Wald	Exp (B)	Exp (B) 95%CI
Gender	-0.16	0.03	23.82	0.86***	(0.81 : 0.91)
Age	0.02	0.00	221.48	1.02***	(1.02 : 1.02)
SES	-0.23	0.02	153.87	0.79***	(0.76 : 0.82)
Rural	0.76	0.04	316.89	2.14***	(1.97 : 2.33)
Income	0.00	0.00	7.12	1.00**	(1.00 : 1.00)
Time	-0.38	0.20	3.80	0.68	(0.47 : 1.00)
Attitude	-0.13	0.03	26.33	0.88***	(0.84 : 0.92)
Time ✖ Attitude	0.04	0.01	8.94	1.04**	(1.01 : 1.07)
Constant	0.79	0.38	4.29	2.20*	

*** = *p* < .001,

** = *p* < .01,

* = *p* < .05.

**Fig 4:**
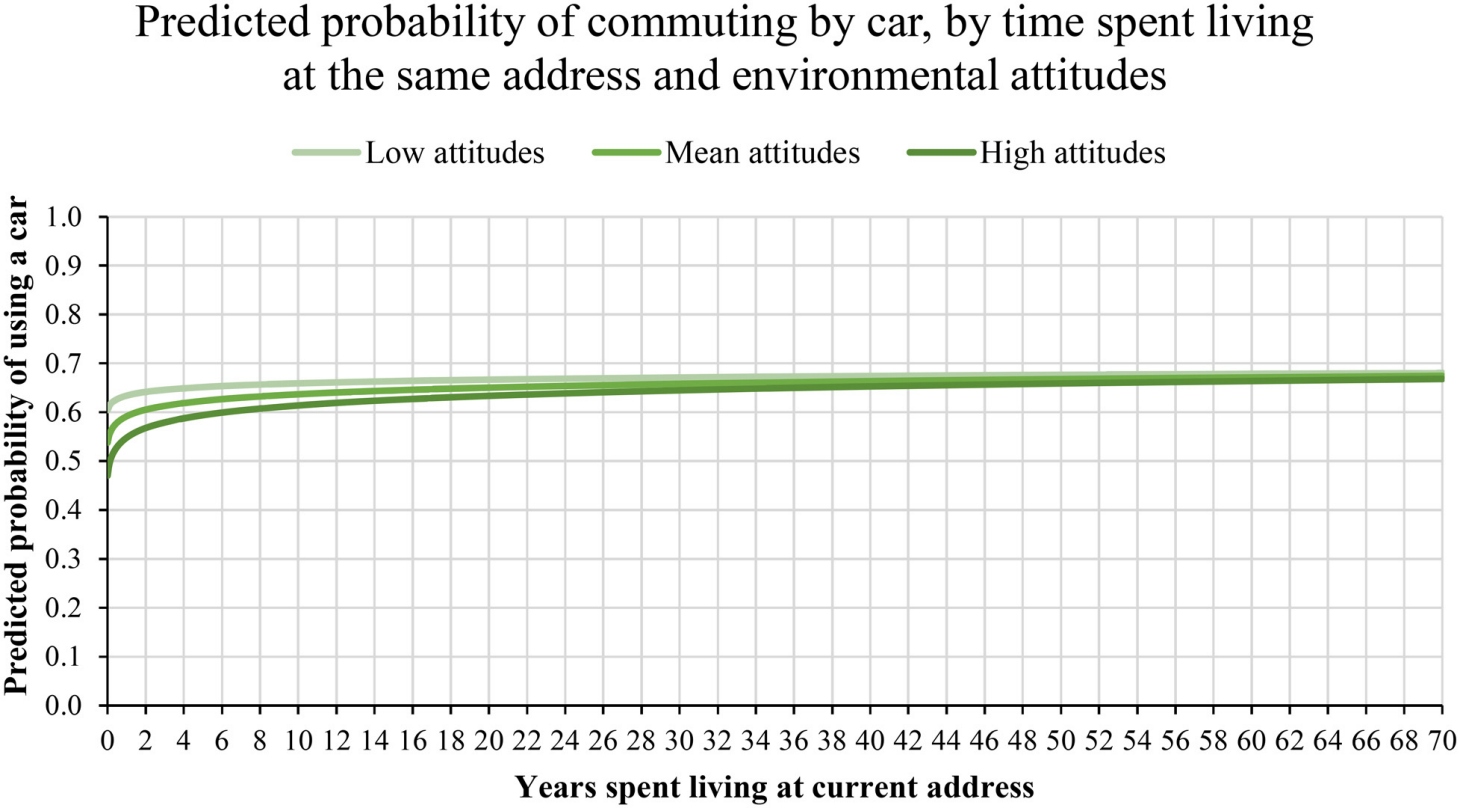
Predicted probability of commuting by car, by interaction between time spent living at the current address and mean and ±1SD of environmental attitudes, controlling for mean values of covariates. Time was log10 transformed for analysis, and displayed here in the original metric for interpretation.

Although a significant link between environmental attitudes and commuting behaviour is found, the interaction between attitudes and time spent living at the same address is significant, which requires interpretation. To illustrate an interaction between two continuous variables, it is advised that researchers separate one continuous variable into three levels, to demonstrate how variance in one variable interacts with other variables [33]. Splitting a continuous variable for illustration is typically performed by indicating the mean value of the variable, as well as -1 SD and +1SD of the same variable. We selected environmental attitudes for this comparison, which provides an indication of approximately ‘low’ (i.e., -1 SD of the mean) ‘medium’ (i.e., mean attitudes), and ‘high’ (i.e., +1 SD of the mean) environmental attitudes. As described earlier, we used the regression equation to calculate the log odds of commuting by car, holding demographic covariates constant at their mean, and allowing the coefficient for Time to vary. To include attitudes in the equation, we created three regression equations that set covariates at their mean, allowed the coefficient of time to vary, and set the coefficient of Attitude as a constant of 1SD lower than the mean of attitudes (“Low attitudes”), constant at the mean of attitudes (“Mean attitudes”), or constant at 1SD higher than the mean of attitudes (“High attitudes”). These three sets of log odds were then calculated as predicted probabilities and plotted, as seen in Fig 4.

As shown in Fig 4, for people who have spent only a small amount of time at their current address, stronger environmental attitudes are linked to a lower probability of car use, and weaker environmental attitudes are linked to higher probability of car use. After living at the same address for 1 month, the predicted probability of commuting by car for people with low environmental attitudes (controlling for covariates at their mean value) is .60, but the predicted probability of commuting by car for people with strong environmental attitudes is .47. However, this effect is relatively short-lived, and a sharp rise in probability of car use is observed as the time spent living at the same location increases, before gradually increasing over time. Again holding covariates constant at their mean, after living in the same address for 12 months, people with low environmental attitudes have a predicted probability of .63, and those with strong attitudes have a predicted probability of .55 for commuting by car. Notably, the difference in probability of car use, while clear immediately after moving home, appears to have little effect over longer periods of time. Living at the same address for 10 years, the predicted probability of commuting by car for people with low environmental attitudes is .66, compared with predicted probability of commuting by car for people with high environmental attitudes of .61, and these differences become increasingly smaller as the time spent living at the same location increases. The results therefore support Hypothesis 2, where environmental attitudes are a significant predictor of commute travel mode choice, but only after recently moving to a new residence.

## Discussion

This paper explores whether commuting by car is lower among people with stronger pro-environmental attitudes after recently moving home, where context change would weaken previous travel mode habits. Using the Understanding Society Survey (USS) with data from over 18,000 respondents, two main results are reported. First, we demonstrate that the length of time a person has lived at their current address is linked to their choice of travel mode for commuting to work. Results show that the predicted probability of commuting by car is lowest after recently moving to a new home, then sharply increases as the time spent living in one location increases between 0 and 24 months, before showing a slow and gradual increase in probability as the time spent living at the same address increases over several years. Second, we included environmental attitudes as a predictor, along with an interaction effect with time at current address. Results show a significant interaction, where stronger environmental attitudes are linked to lower levels of car use immediately after moving home, but that this link between attitudes and behaviour diminishes over time. The results thus support the habit discontinuity and self-activation hypothesis [15], where environmental attitudes are predictive of travel mode choice behaviour, but only after a recent break in local context.

### Results support previous work on habits

Habits are automatic responses to contextual cues in the environment [6]. This automaticity conflicts with behavioural intentions, so that when habits are stronger, intentions are less predictive of behaviour [7–9]. Crucially, the link between the context of a behaviour and the activation of the habit opens the potential for reducing the influence of habits, particularly when moving home to a new location. Previous work has demonstrated that a change in the context of task can increase awareness of new options [16,17], and that travel mode choice habits are weakened when changing travel mode destination [19]. Moving home also requires consideration of practicalities such as how to travel to work: 87% of UK households reported considering travel issues when moving home [34]. Qualitative research into the experience of moving home suggests this consideration involves a complex set of influences to determine how to commute to work, such as instrumental demands and personal preferences [35]. Given that the current study finds that environmental concern is predictive of lower car use, but only after recently moving home, it is therefore likely that people’s habits were weakened by moving home which allowed greater consideration of their views when making travel decisions.

The results from this study thus support the conclusions by Verplanken et al. [15]. With a breaking of previous habits and increased consideration of travel mode choice after moving home, the self-activation hypothesis would suggest that pro-environmental views become a stronger influence on decisions of travel mode choice. Additionally the inverse is also supported; people who resided at the same location for longer periods of time (a more stable context) had negligible links between their pro-environmental views and their travel mode choice behaviour. The lack of association between attitudes and behaviour is likely to be reflective of the weaker link between intentions and behaviour when habits are stronger [9], and stronger travel habits minimise the impact of conscious intentions on travel mode choice [11].

### Replication and impact for future interventions

The original proposal by Verplanken et al. [15] may be have been influenced by the select sample at one UK University, particularly socio-economic or geographical factors that affect travel mode decisions [23,24]. The current results arise from a large and geographically diverse sample, whilst controlling for differences in age, gender, monthly income, socio-economic status, and urban/rural location. There is now strong evidence to support the habit discontinuity and self-activation hypothesis effect on travel mode choice after moving home. There has been some controversy over the replicability of several social psychology findings [25,26], and we hope that the current results instil more confidence into the phenomenon of context changes upon habits.

The observed effects are of conventionally “small” size. Some guidelines suggest that odds ratios of 1.68, 3.47, and 6.71 respectively, are analogous to Cohen’s *d* values of .2, .5, and .8; the suggested thresholds of “small”, “medium”, and “large” effect sizes [36]. Analysis 1 gave an odds ratio of 1.23 for time spent living in the same location to predict commute travel mode choice, and the interaction between environmental attitudes and time at current location gave an odds ratio of 1.04. However two points should be considered. First, given the large volume of emissions generated by transport [1], even a small reduction in the proportion of people commuting by car could have a sizeable positive effect in reducing greenhouse gas emissions, and is worth considering further. Second, this analysis does not evaluate a carefully prepared intervention or experiment; it is evidence that a natural break in context encourages behaviour (albeit temporarily) to be more in line with a person’s attitudes, and may facilitate behaviour change interventions. Life-course changes have an impact on travel mode choices which offer opportunities for interventions [21,22], and others have highlighted home relocation as a time where people may be more receptive to information campaigns on travel decisions [37]. Habitual behaviours require less information for people to make decisions [12,13], which makes conventional information-based campaigns less effective at habitual behaviours [9,14]. The natural break of home relocation therefore offers an excellent opportunity to directly target and capitalise on this shift. If such sustainable behaviour changes are observed naturally, giving people additional information and motivation at the time of moving home could yield even greater benefits, and further research or practice should explore this concept.

### The declining influence of contextual changes

The USS dataset also allowed analysis of commute decisions over long periods of time since moving home. In Analysis 2, plotting the predicted probability of commuting by car for ‘low’, ‘medium’ and ‘high’ environmental attitudes suggests the largest influence of attitudes is seen between 0 and 24 months since moving home. The original proposal by Verplanken et al. [15], suggested that a period of 12 months may be a suitable period to evaluate the habit discontinuity effect, at least for effects on travel mode use after moving home. The present results broadly support this concept, which would indicate that interventions could therefore focus on delivering resources within the first 12 months of moving home. However, we find that the strongest differences in predicted probability were observed in the very early months after moving home and soon decreased over time, and so our results suggest delivering resources as soon as possible to capitalise on this natural break in context.

It is interesting to observe that, in general, the predicted probability of commuting by car is lower (around .50) immediately after moving home, and rising until a stable level of around .67 as the time since a home move increases. It is not immediately clear why car use is reduced after moving home, even for respondents with low environmental concern, which is a topic that could be explored in more detail. Notably, people with strong environmental attitudes show a significantly greater reduction in car use after moving home, but also demonstrate a steady rise in the predicted probability of commuting by car the longer they reside at their current address. It may be that despite an initial preference for avoiding car use, people experienced difficulties in forming sustainable travel habits.

Only a limited number of studies have explored how habits form, and results suggest the process is widely variable and not fully understood. Lally and colleagues [27] attempted to model the daily habit formation of various health behaviours (e.g., drinking water, sit-ups), but encountered several issues; some participants showed no habit growth, and some growth curves could not be modelled. For the habit growth curves that were applicable, the median time taken to develop a stable habit was 66 days (approx. 2 months), but the range of time to develop stable habits varied greatly from between 18 days to 254 days: approximately 8½ months [27]. Commuting to work arguably involves more complexity than brief health behaviours, and so the potential for external influences to disrupt formation of a non-car travel habits may be even greater, but it is not clear how this may occur. Additional research is required to track the formation of habitual behaviours over periods of time, which may identify and develop strategies to encourage the durability of sustainable habits over time.

### Strengths and weaknesses of the study

Although a validation of previous research with a large and representative dataset, this analysis has some aspects for critical consideration. Environmental attitudes, measured with nine yes/no statements, offers a limited range of scores, and has not been validated as well as more established measures of pro-environmental views. Developments in predicting pro-environmental behaviour have explored alternative methods, such as using value orientations based on Schwartz’s [38] circumplex model. In particular, the use of biospheric or egoistic values measured in De Groot and Steg’s [39] value orientation measure, has shown reliable use in predicting pro-environmental behaviour above conventional measures of environmental worldviews [40,41]. Using more robust and validated measures of environmental concern may be more effective at uncovering links between personal views and behaviour. Additionally, we note that the sample had 9.1% of cases with missing data for environmental attitudes, and cases with missing data were more likely to be older, higher socio-economic status, and higher earners than those who took part. Despite the strength and size of the sample analysed, results must also consider that there may be some bias in responses of environmental concern.

Although using a large sample size with a representative sample, the analysis used cross-sectional data which cannot account for possible differences in commute mode choice prior to moving home. Similarly, the analysis controlled for the effects of age in the regression models, but it cannot be ruled out that age was not a factor in the identified relationship between travel mode choice and time living at current location, as greater time spent at one location necessitates being of an older age. People who regularly move home may be likely to be younger people, who may be more inclined to act on environmental views. Alternatively, older people’s priorities may change, whereas environmental concern predicted use of alternative travel modes when younger, increased concerns for health may make older people more dependent on car use (as opposed to physically active travel modes) despite these attitudes. The replication of the interaction between time since moving home and attitudes is encouraging, but future work should consider longitudinal analyses into behaviour changes as people move home for a more in-depth evaluation.

Categorisation of travel mode choice was also quite limited, with respondents choosing a preferred main mode of travel to work but not accounting for individual variations in travel mode choice. For example, a person may drive 3 days a week, choose to cycle for 2 days for environmental reasons, but could still be counted solely as a car user. Travel routines can be a mix of mode choices, and more sophisticated measures of travel mode preferences may yield a more detailed relationship between changes in context and travel mode choice. Additionally, the intricacies of motivations and reasons for changing behaviour are not evident in this analysis. These results would be well-served by additional qualitative investigations into transport demands after moving home, particularly longitudinal work exploring whether pro-environmental concern does have a greater weight in transport decisions after relocation. What the current analysis may lack in introspective analysis it makes up for in statistical power, but a combined methodological approach would help to further answer these questions.

## Conclusions

In conclusion, this article reports an investigation of Verplanken et al.’s [15] habit discontinuity and self-activation hypothesis using a nationally-representative sample of the UK population. Analysing time since moving home, pro-environmental attitudes and commute travel mode choice, we find two main results. First, we find that independent of age, gender, socio-economic status, monthly income, and urban or rural location, the length of time living at a current address is linked to commuting travel mode choice; car use is lowest after recently moving home, and then increases in probability over time. Second, by adding environmental attitudes, we find that strong environmental attitudes predict lower probability of commuting by car, but this effect is only observable after recently moving home, and that the link between environmental attitudes and travel mode choice grows weaker as people live in the same residence for longer periods of time. We conclude that moving home weakens habits and encourages greater consideration of one’s on views, but only for a short period. Interventions should target those who have recently moved home in order to capitalise on this window of opportunity.
